# Ruptured and infected pelvic hydatid cyst with peri-appendiceal inflammatory involvement in an adolescent: laparoscopic management – a case report

**DOI:** 10.1097/RC9.0000000000001018

**Published:** 2026-09-23

**Authors:** Hela Oueslati, Aida Daib, Souha Laarif, Cyrine Saadi, Rabiaa B. Abdallah, Youssef Hellal

**Affiliations:** a Faculty of Medicine of Tunis, University Tunis El Manar, Tunis, Tunisia; b Department of Pediatric Surgery, Habib Thameur Hospital, Tunis, Tunisia

**Keywords:** adolescent, case report, laparoscopy, pelvic hydatid cyst, periappendicitis

## Abstract

**Introduction:**

Pelvic hydatid disease is uncommon, particularly in pediatric patients, and may mimic abdominal or gynecological conditions. Acute complications, such as rupture and secondary infection, may result in challenging diagnostic presentations and surgical emergencies.

**Presentation of case:**

We report a 15-year-old girl presenting with acute abdominopelvic pain and vomiting. Initial imaging revealed a large preuterine cystic mass (10.6 cm) without a clear digestive or adnexal origin, alongside a small hepatic cystic lesion. Given its presumed gynecologic origin and the delay in magnetic resonance imaging access, the patient was referred for further evaluation but was lost to follow-up. Three weeks later, she was readmitted with fever, worsening pain, and elevated inflammatory markers. Emergency laparoscopy revealed a ruptured, infected preuterine hydatid cyst containing purulent fluid and daughter membranes, adherent to the uterus and right adnexa. An inflamed appendix with marked peri-appendiceal changes was found in close contact with the cyst, suggesting peri-appendiceal inflammatory involvement secondary to local inflammatory extension. Laparoscopic management included cyst evacuation with scolicidal precautions, appendectomy, cavity drainage, and peritoneal lavage. The hepatic cyst was managed conservatively with a 3-month course of albendazole. No recurrence was observed at 1-year follow-up.

**Discussion:**

Pelvic hydatid disease is rare and diagnostically challenging. Rupture and superinfection may obscure the diagnosis. This case highlights peri-appendiceal inflammatory involvement secondary to local extension from an infected, ruptured pelvic hydatid cyst.

**Conclusion:**

Pelvic hydatid cysts should be considered in cystic pelvic masses in endemic areas. In selected cases and with meticulous scolicidal precautions, laparoscopy may be a safe and effective therapeutic approach.

## Introduction

Hydatid disease, caused by *Echinococcus granulosus*, remains a major parasitic health problem in endemic regions, with significant morbidity despite ongoing control efforts^[^1^]^. The liver and lungs are the most commonly affected organs, whereas pelvic involvement is rare, particularly in children and adolescents^[^2,3^]^.

HIGHLIGHTS

- Pelvic hydatid disease should be considered in the differential diagnosis of cystic pelvic masses.
- Rupture and superinfection may obscure the initial diagnosis.
- Secondary appendiceal inflammation may occur by contiguous spread of inflammation.
- Careful scolicidal precautions facilitate safe laparoscopic management.
- Laparoscopy allows the simultaneous treatment of associated abdominal pathology.

Clinical presentation is often nonspecific. Symptoms may evolve slowly due to mass effect^[^4^]^ or appear acutely when complications such as rupture or secondary infection occur^[^5^]^. These complications can rapidly transform a previously indolent lesion into a surgical emergency.

Peri-appendiceal inflammatory involvement secondary to local inflammatory extension from a ruptured pelvic hydatid cyst appears to be uncommon and remains rarely discussed in the literature. Recognition of such atypical presentations is important for appropriate surgical management.

We report on a 15-year-old girl with a ruptured and infected pelvic hydatid cyst and peri-appendiceal inflammatory involvement secondary to local inflammatory extension, which was successfully managed with laparoscopic surgery.

This case report has been reported in line with the SCARE checklist^[^6^]^.

## Case presentation

A 15-year-old girl presented with acute lower abdominal pain and repeated vomiting for 24 hours (Table 1). She had no prior medical or surgical history and no known exposure to hydatid disease. She lived in an urban area within an endemic region.

**Table 1:** Timeline of clinical events.

Date/time point	Clinical findings	Investigations	Management
Initial presentation	Lower abdominal pain and vomiting	WBC 10 620/mm^3^; CRP 20 mg/L; US and CT showing 10.6-cm pelvic cyst and 4-cm hepatic cyst	Surgical emergency excluded; MRI and gynecological evaluation planned
Following 3 weeks	Lost to follow-up because of delayed MRI access	No reassessment	No treatment
Emergency readmission	Fever (38.5°C), worsening abdominal pain, right iliac fossa tenderness	WBC 15 000/mm^3^; CRP 120 mg/L	Emergency laparoscopy
Surgery	Ruptured infected pelvic hydatid cyst with daughter membranes and peri-appendiceal inflammatory involvement	Histopathology confirmed hydatid disease and periappendicitis	Laparoscopic cyst evacuation, appendectomy, drainage
Follow-up	Asymptomatic	Serial ultrasonography	Albendazole for 3 months
One year	No recurrence	Hepatic cyst regression from 4 cm to 2 cm	Continued surveillance

On examination, she was afebrile and hemodynamically stable. Abdominal palpation revealed tenderness in the lower abdomen without guarding or rebound. Initial laboratory investigations showed a white blood cell count of 10 620/mm^3^, a C-reactive protein level of 20 mg/L, and a negative β-hCG test.

Ultrasonography revealed a large pelvic cystic mass and a small, well-circumscribed unilocular hepatic cystic lesion with anechoic content and posterior acoustic enhancement. Based on its morphological appearance, the lesion corresponded to a CE1-type cystic pattern according to the WHO-IWGE classification of cystic echinococcosis, although its exact etiology was not established at that stage.

Contrast-enhanced computed tomography (CT) (Fig. 1) confirmed a well-circumscribed preuterine cyst measuring 10.6 cm, with no clear digestive or adnexal origin, and demonstrated a small hypodense lesion in hepatic segment III. No imaging signs of appendicitis were detected.

**Figure 1.**
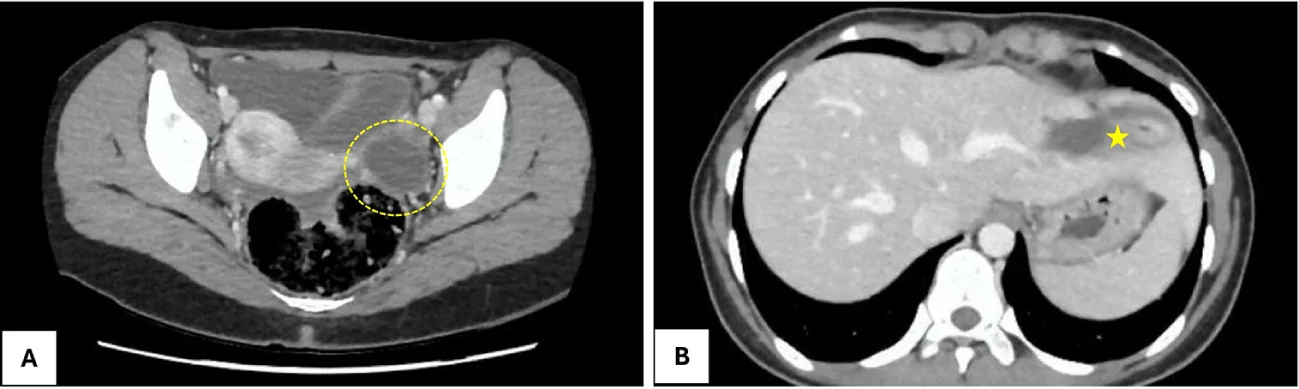
Contrast-enhanced computed tomography (CT) of the abdomen and pelvis: (A) Axial pelvic CT image showing a well-circumscribed cystic lesion in the preuterine pelvic region (dashed circle), measuring approximately 10.6 cm in maximal diameter, without definitive features to determine its origin. (B) Axial abdominal CT image demonstrating a small hypodense cystic lesion in hepatic segment III (star), suggestive of a concomitant hepatic cyst.

Initial differential diagnoses encompassed ovarian, paraovarian, Müllerian, and mesenteric cysts, as well as hydatid disease. Given the absence of pathognomonic imaging features and the lack of a definitive association with the incidental hepatic cyst, pelvic magnetic resonance imaging (MRI) and gynecological consultation were requested for further characterization. However, delayed MRI access and loss to follow-up precluded timely evaluation until an emergency readmission 3 weeks later, with clinical deterioration.

She returned with worsening pain, a fever of 38.5°C, and localized right iliac fossa tenderness. Laboratory tests showed leukocytosis (15 000/mm^3^) and an elevated C-reactive protein level (120 mg/L).

Given clinical deterioration and limited imaging availability, emergency laparoscopy was performed using a three-port technique: a 10-mm umbilical trocar for the laparoscope and two 5-mm trocars in the right and left iliac fossae. Intraoperatively, a small, uncomplicated hepatic hydatid cyst was noted in segment III (Fig. 2A). The pelvis contained a ruptured pre-uterine hydatid cyst approximately 10 cm in diameter (Fig. 2B), with purulent material and daughter membranes. Dense adhesions involved the uterus and right adnexa (Fig. 2C). The appendix appeared inflamed but non-perforated (Fig. 2D) and was firmly adherent to the infected cyst, raising suspicion of peri-appendiceal inflammatory involvement secondary to local inflammatory extension.

**Figure 2.**
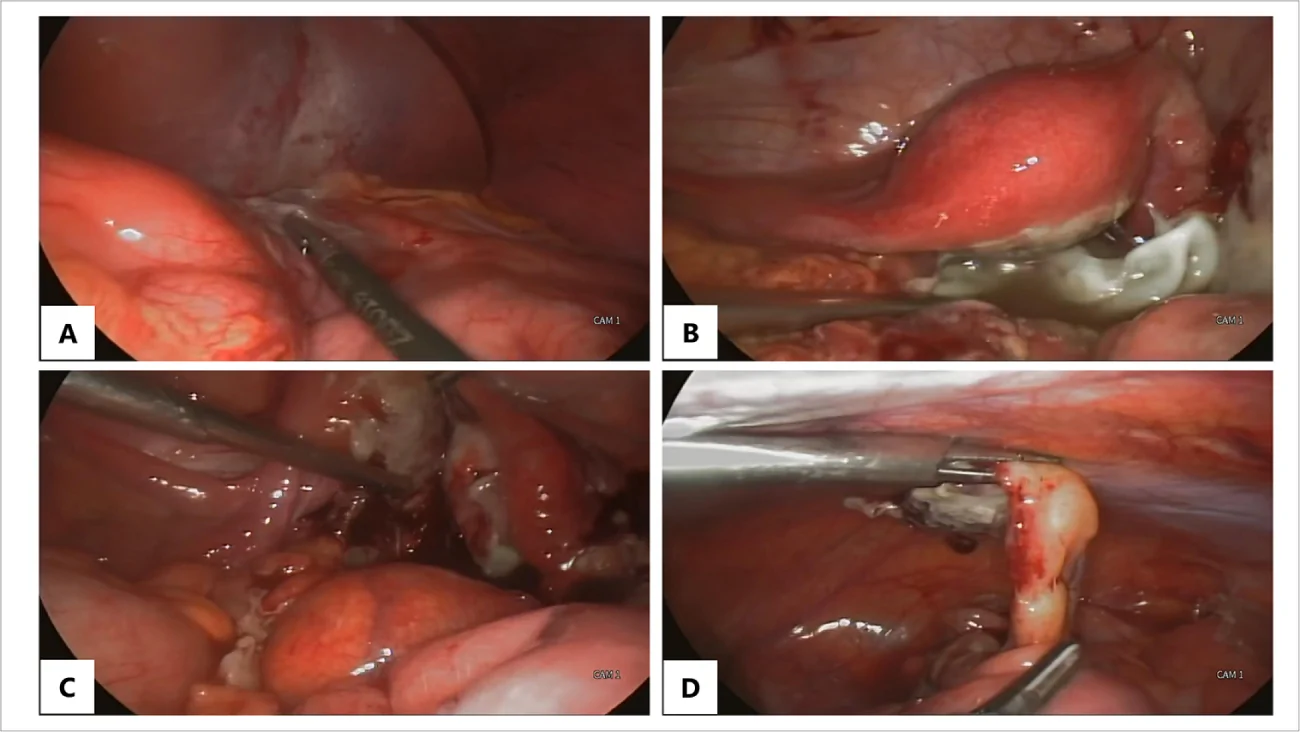
Intraoperative laparoscopic views of: (A) A small, uncomplicated hepatic hydatid cyst measuring approximately 4 cm, located in segment III of the liver. (B) A ruptured and infected pre-uterine hydatid cyst measuring approximately 10 cm, containing purulent fluid and multiple daughter membranes. (C) A pelvic hydatid cyst adherent to the uterus, right ovary, and right fallopian tube, demonstrating dense inflammatory adhesions in the pre-uterine space. (D) An inflamed, non-perforated appendix in close contact with the infected pelvic hydatid cyst, consistent with peri-appendiceal inflammatory involvement by contiguity.

To minimize dissemination, the operative field was isolated with pads soaked in 20% hypertonic saline. Following cyst rupture, the residual cavity – containing purulent fluid and daughter membranes – was aspirated, instilled with 20% hypertonic saline as a scolicidal agent for 10 minutes, and then re-aspirated. Daughter membranes were retrieved with atraumatic graspers. The appendix and parasitic membranes were extracted en bloc via an endoscopic retrieval bag through the umbilical port. A 10-Fr Redon drain was placed within the residual cavity. Given intraperitoneal spillage of purulent and parasitic material, thorough peritoneal lavage was performed.

Postoperatively, the patient received amoxicillin-clavulanate, guided by the antibiogram of the intraoperative peritoneal fluid culture, for a total of 10 days (5 days intravenously followed by a 5-day oral course), and the recovery was uneventful. A chest radiograph was performed to rule out associated pulmonary hydatid disease and revealed no abnormalities. Hydatid serology performed postoperatively was positive and served as a baseline parameter for postoperative follow-up. The patient was discharged on postoperative day 5 with albendazole therapy (10–15 mg/kg/day) prescribed for 3 months. Histopathology confirmed hydatid disease and periappendicitis with parasitic membranes in the mesoappendix.

The hepatic lesion was classified as a WHO-IWGE CE1 cyst. Given its small size, uncomplicated appearance, and the emergency surgical setting, medical treatment with albendazole and radiological surveillance were preferred.

Follow-up included abdominal ultrasonography every 3 months and monthly laboratory tests, including leukocyte counts and aminotransferase measurements, to detect adverse reactions to albendazole.

At 1-year follow-up, the patient remained symptom-free, with regression of the hepatic CE1 cyst from approximately 4 cm at baseline ultrasonography to approximately 2 cm during follow-up (Fig. 3) and no evidence of pelvic recurrence.

**Figure 3.**
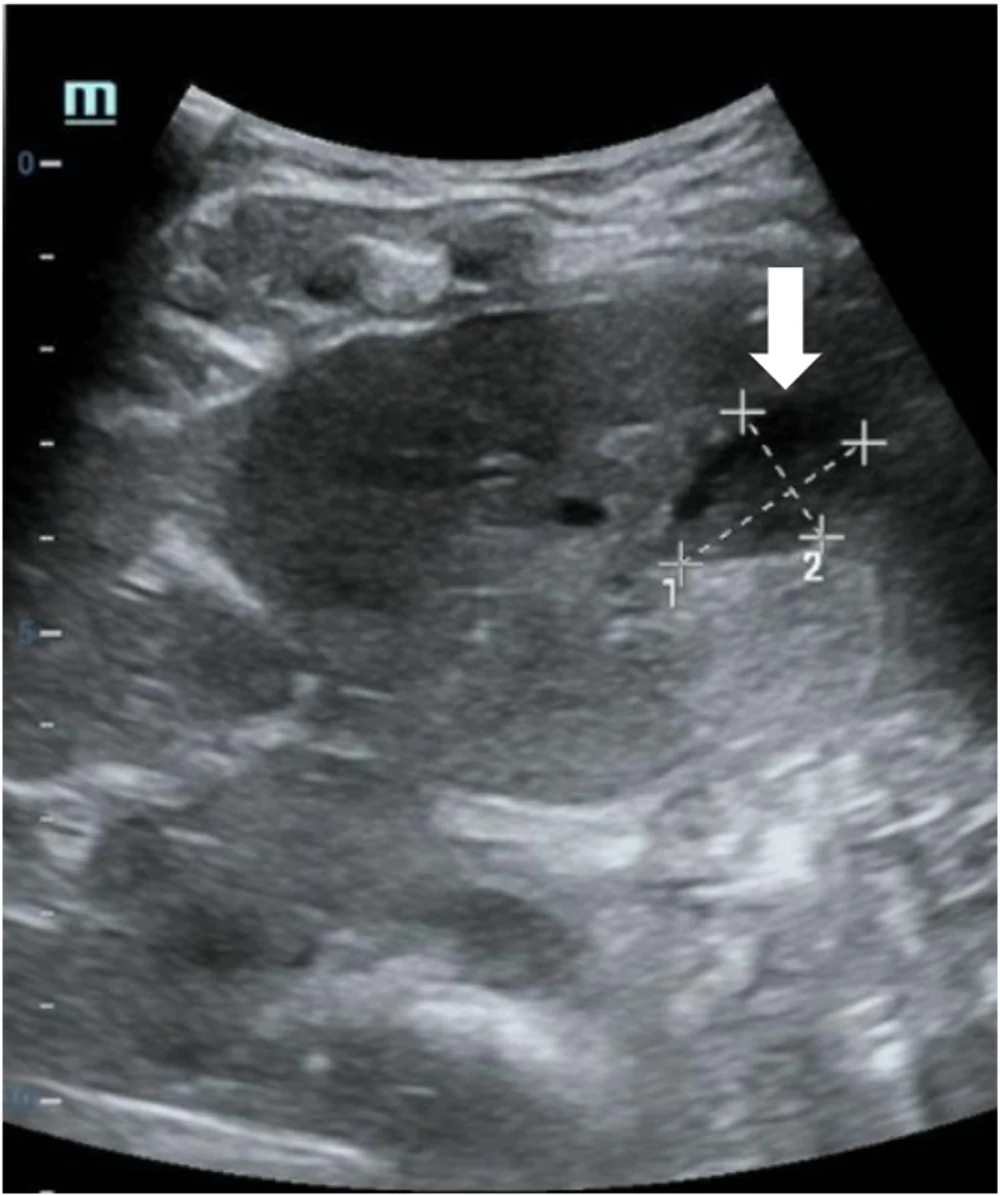
Follow-up ultrasonography showing a residual hepatic CE1 hydatid cyst in segment III (White arrow) with significant size reduction during follow-up.

## Discussion

Pelvic hydatid disease is an uncommon manifestation of cystic echinococcosis, representing approximately 0.2–0.9% of reported cases^[^2^]^ and is particularly rare in children and adolescents^[^3^]^. Cystic echinococcosis remains a significant zoonotic parasitic disease in endemic regions, with substantial morbidity despite control efforts^[^1^]^. In most instances, pelvic involvement is considered secondary to peritoneal dissemination following rupture of a hepatic or splenic cyst, although true primary pelvic localization has been described, possibly related to hematogenous or lymphatic spread^[^7,8^]^.

In female patients, the ovary and uterus are the most frequently involved pelvic organs^[^2,9^]^. Pediatric and adolescent cases remain exceptional, with only isolated reports in the literature^[^3^]^. Importantly, hydatid disease should not be considered exclusively rural, as persistent transmission in urban and peri-urban settings of endemic countries has been documented^[^1,5,10^]^.

Clinical manifestations are often nonspecific and depend on cyst size, location, and complications^[^10^]^. Several pelvic hydatid cysts were initially misdiagnosed as ovarian neoplasms or adnexal torsion due to overlapping imaging features^[^9,11,12^]^. Deep pelvic or retrovesical cysts may present with acute urinary retention, hydronephrosis, or obstructive uropathy^[^8,13,14^]^. Radiological diagnosis may be particularly challenging when classical features such as daughter cysts, detached membranes, or the “water lily sign” are absent^[^15^]^. According to international consensus guidelines, ultrasonography remains the first-line imaging modality, with CT or MRI providing additional anatomical detail in atypical or complicated cases^[^16^]^. In our patient, the pelvic lesion was initially considered more likely to be gynecological in origin, and MRI was requested for further characterization. Delayed MRI access and interruption of follow-up may have contributed to progression toward rupture and secondary infection.

Complicated cysts may rupture spontaneously or secondary to trauma, leading to peritoneal dissemination, secondary bacterial infection, a systemic inflammatory response, or, rarely, anaphylactic reactions^[^5,10,16^]^. Intraperitoneal rupture transforms a chronic parasitic condition into a potentially life-threatening emergency. In the present case, rupture resulted in purulent pelvic contamination and an acute abdomen.

The coexistence of peri-appendiceal inflammatory involvement secondary to local inflammatory extension constitutes the most distinctive feature of this case. Although right iliac fossa pain may occur in pelvic hydatid disease^[^5,10^]^, confirmed appendiceal involvement is exceptionally rare in the literature. The appendix was firmly adherent to the infected cyst; histopathology revealed periappendicitis with parasitic membrane infiltration of the mesoappendix. These findings are consistent with local inflammatory extension from the ruptured hydatid cyst, though a definitive causal relationship cannot be established. Accordingly, the appendiceal involvement is best interpreted as contiguous peri-appendiceal inflammation rather than primary acute appendicitis. Such secondary inflammatory phenomena have been described in peritoneal echinococcosis, where adjacent organ involvement may occur through direct contact and local inflammatory reaction^[^4,8,17^]^.

Surgical management remains the cornerstone of treatment, aiming for complete evacuation of parasitic material, prevention of spillage, and avoidance of secondary dissemination^[^2,16^]^. Historically, laparotomy was preferred for complicated or ruptured cysts. However, with increasing experience, laparoscopy has emerged as a feasible and safe approach in selected patients when strict precautions are observed, including isolation of the operative field and the use of scolicidal agents^[^2,11^]^. Concerns persist regarding pneumoperitoneum-related dissemination^[^2^]^, yet accumulated evidence suggests that minimally invasive surgery offers reduced postoperative pain, shorter hospital stays, and faster recovery without increasing recurrence rates when performed by experienced teams^[^16^]^.

Adjunctive albendazole therapy plays a critical role in reducing recurrence risk and managing small residual or concomitant cysts^[^16^]^. Current recommendations suggest administration at 10–15 mg/kg/day for several weeks to months, depending on cyst stage and surgical findings^[^16^]^. In our case, postoperative albendazole for 3 months was associated with marked regression of the small hepatic lesion and no recurrence at 1-year follow-up.

The value of this case lies not only in its unusual pelvic location but also in the diagnostic challenge posed by the coexistence of pelvic and hepatic cystic lesions, the delay in definitive characterization, the occurrence of rupture and secondary infection, and the successful laparoscopic management with reproducible scolicidal precautions.

## Conclusion

Pelvic hydatid disease should be considered among the differential diagnoses of cystic pelvic masses in adolescents living in endemic regions. Rupture and secondary infection may mimic more common surgical or gynecological emergencies. This case also illustrates that peri-appendiceal inflammatory involvement may occur through local extension from an infected, ruptured pelvic hydatid cyst. When meticulous scolicidal precautions are respected, laparoscopy may be a safe and effective therapeutic approach even in complicated presentations.

## Data Availability

The data supporting the findings of this case report are not publicly available due to patient privacy and confidentiality concerns. Relevant clinical information is included within the manuscript.
